# Effects of dietary yeast beta-1,3-1,6-glucan on growth performance, intestinal morphology and chosen immunity parameters changes in Haidong chicks

**DOI:** 10.5713/ajas.18.0962

**Published:** 2019-03-07

**Authors:** Baoan Ding, Juanshan Zheng, Xiezhong Wang, Licheng Zhang, De Sun, Quanhu Xing, Andrea Pirone, Baldassare Fronte

**Affiliations:** 1 State Key Laboratory of Plateau Ecology and Agriculture, College of Agriculture and Animal Husbandry, Qinghai University, Xining 810016, China; 2 Animal Disease Prevention and Control Center of Qinghai Province, Xining 810001, China; 3 Animal Husbandry and Veterinary Station of Huzhu County of Qinghai Province, Huzhu 810500, China; 4 Department of Veterinary Science, University of Pisa, 56124, Pisa, Italy

**Keywords:** Body Growth, Haidong Chicks, Intestine Morphology, Yeast, Dietary Beta-glucan

## Abstract

**Objective:**

This study investigated the effects of 1,3-1,6 beta-glucan added to the diet of Haidong chicks reared under hypoxic conditions, to ascertain the growth performances, immunity and intestinal morphology changes.

**Methods:**

A total of 750 chicks were divided into five groups and fed diets containing 0.5 g/kg, 1.0 g/kg, and 2.0 g/kg 1,3-1,6 beta-glucan from yeast (G1, G2, G3, respectively), 0.2 g/kg Taylor rhizomorph and a control feed.

**Results:**

The body weight and body weight gain were higher in chicks fed 1,3-1,6 beta-glucan and Taylor rhizomorph than in control group. Feed conversion ratio significantly differed for G2 and G3 groups in comparison to control group. The relative weight of bursa was higher in G1, G2, and G3 groups. The white blood cells and lymphocytes were significantly increased in groups fed 1,3-1,6 beta-glucan. The immunoglobulin G of serum peak appeared in the G3 group. The villous height of the duodenum was higher in 1,3-1,6 beta-glucan feed groups. In the jejunum, the villous height was higher in G2 and G3 groups and crypt depth for all the groups fed β-glucan. At ileum level the villous height and crypt depth was higher for groups G1, G2, and G3.

**Conclusion:**

The growth performance of Haidong chicks is improved when 10 and 20 g/kg 1,3-1,6 beta-glucan is included in the diet; hence, it is suggested that 1,3-1,6 beta-glucan be included in poultry diet to reduce and replace the use of antibiotics.

## INTRODUCTION

In the poultry industry, antibiotics have been widely used to protect birds from the adverse effects of pathogenic and non-pathogenic enteric microorganisms and improve growth performance and production. However, problems such as antibiotics resistance of pathogens and antibiotic residues in poultry products are frequent consequences of the repeated use of antibiotics in poultry diets [1]. To address this problems, several international institutions such as WHO, FDA, EFSA, and others, have strongly asked to ban or reduce the use of antibiotics in animal production. Hence, to maintain broilers health and an efficient production, feed manufacturers and research communities have proposed several alternatives to the broiler industry [1,2]. Among these, β-glucans have received considerable attention because of their promoting growth and immunomodulatory effects [3]. β-Glucans are polymers of glucose extracted from yeast cell walls (YCW), bacteria, fungi and cereals such as oats and barley. β-Glucans from yeast and fungi consist of a backbone of glycopyranosyl molecules joined by 1,3-β-links; from this backbone, side chains can be joined by 1,6-β-links [4], producing a branched molecular structure. Fungal β-glucans have short branches while they are long in yeast. On the other hand, bacterial β-glucans are unbranched with only 1,3-β-linkages between the glycopyranosyl molecules; similarly, barley and most cereal grains contain unbranched β-glucan chains [5].

β-Glucans have drawn ever-increasing attention thanks to their innate immune system immunomodulatory properties [6]. The 1,3-1,6 β-glucan present in YCW should be recognized as an immune modulator substance in animals and humans [7]; thus, dietary YCW might exert some benefits on the immune system of intestinal mucosa [8]. It has been reported that supplementation of broiler diets with yeast-derived products improve body weight (BW), body weight gain (BWG), and feed conversion ratio (FCR) by establishing beneficial microflora in the gastrointestinal tract, and facilitating gut development [9,10]. However, other studies demonstrated no significant difference in growth performance parameters [11–13]. Dietary yeast β-glucan supplementation for chicks resulted in higher villus height and villus height to crypt depth ratio than for the unsupplemented chicks [3,14]. The diet supplementation with β-glucan significantly increased the number of goblet cells in the jejunum of chicks [3], this suggesting that β-glucans promote gut health [15,16].

The Haidong chick is an ancient breed of chicken from the Qing-Tebit plateau of China. It is notable for its unusual double or rose comb, for the late age at which birds reach maturity, and for the small size and colour of the eggs. The ear-lobes are white. Average weight is 1.6 to 1.8 kg for cocks, 1.3 to 1.4 kg for hens. The eggs range from white to light brown in colour, and weight at least 50 g. The quantity of egg production is 160 per year in natural range. It is adapt to live in semi-agricultural and semi-pastoral ecological environment in the alpine. Hence, the objective of the present study was to investigate the dietary effects of 1,3-1,6 β-glucan on growth performance, immunity and the intestinal morphology in Haidong chicks raised in high altitude area.

## MATERIALS AND METHODS

### Birds management and treatments

The experiment was carried-out in Xueling Haidong Chicken Breeding Farm, Huzhu County, Qinghai, China (36° 42′ 37″N, 102° 10′ 57″E; the altitude is 3,650 meter), according to a protocol approved by Qinghai University Animal Care Committee.

Haidong is a typical local chicken breed of Qinghai province, China. A total of 750 one-day old Haidong mix-sex chicks were randomly assigned to 5 dietary treatments and 5 replicates, with 30 birds per replicate. The experimental design consisted in a negative control group (C, basal diet), and positive control group (T, basal diet with 0.2 g/kg Taylor rhzomorph. Taylor rhzomorph was used in feed of chicks in Farm of Haidong chicks as antibiotics) and three treated groups (G1, G2, G3, basal diet with 0.5, 1.0, and 2.0 g/kg 1,3-1,6 β-glucan, respectively); to this purpose, a 80% pure commercial product extracted from *Saccharomyces cerevisiae* cell wall (Nanjing Elegant Nutraceuticals Co., Ltd. Nanjing, China), was used as source of 1,3-1,6 β-glucan; the details of the diets are shown in Table 1.

They were kept in chicken battery cages (3-layer, 30 birds/cage). Diets were fed in mash form, and feed and water were provided *ad libitum* throughout the experiment. The photoperiod were 22 L:2 D from 0 to 3 days, 20 L:4 D from 3 days to 1 week, 18 L:6 D from 1 to 2 week, 16 L:8 D from 2 to 3 week, 14.5 L:9.5 D from 3 to 6 week. The current study lasted 6 wk. The temperature inside the experimental room was maintained between 31°C± 3°C from 1 to14 d and at 26°C± 3°C from 15 to 42 d of age, and the mean daily humidity was 55%±2%. The chicks were raised up to 42 days of age. On day 21st and 42nd, BW and feed intake (FI) were measured; hence BWG and FCR of the periods 0 to 21st and 22st to 42nd day of trial, were also calculated (Table 2). Mortality events were also recorded on a daily bases. Finally, on day 42nd, 3 birds were randomly sampled from each replicate (15 birds per treatment) and sacrificed by cervical dislocation after 4 hours fast; blood samples and other biological samples were also collected as further described.

### Blood profiles

Blood samples were collected from the wing veins into a sterile syringe and divided it in two test tube (one tube is for blood plasma and the other tube for sreums). Blood cell (WBC, white blood cell; RBD, red blood cell; and lymphocytes) were counted using blood plasma. The other tube was immediately centrifuged at 3,000 g for 15 minutes; then, serums were stored in the freezer at −20°C. The concentration of serum immunoglobulin G (IgG), IgM, and IgA were evaluated using a commercially available ELISA kit (Shanghai Enzyme-linked Biotechnology Co., Ltd, Shanghai, China).

### Size of different organs

After cervical dislocation, the sampled birds were manually dissected and weights of the liver, spleen and bursa were recorded and organs relative weight (ratio of weight to final BW) was calculated.

### Histological analysis of the intestinal morphology

Segments of approximately 5 cm of duodenum (midpoint of the pancreatic loop), jejunum (midpoint of jejunum) and ileum (after Meckel’s diverticulum) were obtained before the removal of the entire intestinal tract. Samples were fixed in 4% buffered formalin solution for 24 hours and processed for routine paraffin embedding. Serial 4 μm transverse sections were cut with a microtome (Reichert-Jung. Mod. 1140/Autocut) and collected onto gelatin coated slides and stained with haematoxylin and eosin for morphological evaluation. The sections were examined by a light microscope (Leitz, Diaplan; Leitz, Stuttgart, Germany) connected to a PC via a Nikon digital system (Digital Sight DS-U1; Nikon, Tokyo, Japan). Images were acquired using the NIS-Elements F version 2.10 software (Nikon, Japan). For each bird, two slides, each with a mounted tissue section containing ten well-oriented and intact crypt-villus units were measured in triplicate. The thickness of intestinal wall was defined as the distance from intestinal external subserosa to the muscular junction layer and submucosa. The muscle thickness was measured from the junction between the sub-mucosal and muscular layers to that between the muscular layer and the tunica serosa. The villi height was defined as the distance from villus tip to crypt junction. The crypt depth was defined as the depth of the invagination between adjacent villi. The number of AB/PAS-positive cells along the villi determined the density of cells per mm2. Measurements were made using Image J 1.37V software (National Institutes of Health, New York, USA).

### Statistical analysis

Mean values generated from all individual data were statistically analyzed by a one-factor variance analysis using the general linear model procedure of the SAS Institute (Cary, NC, USA). If the main effect between any groups was found to be significant, they were then compared using the Tukey’s multiple range test at p<0.05.

## RESULTS

After 21 days and 42 days of trial (Table 2) a higher BW, BWG, and FI were observed for chicks fed β-glucan and Taylor rhzomorph in comparison to the negative control group (p<0.05). From 22 to 42 days of trial, a lower (p<0.05) FCR was observed for chicks fed 1.0 g/kg (G2) and 2.0 g/kg (G3) β-glucan in comparison to control group (C), while differences were not statistically significant in comparison to the other considered groups (G1 and T). Moreover, the highest mortality rate was observed in the negative control group and the lowest in chicks fed 2.0 g/kg β-glucan (G3) and positive control group (T), even though differences were not statistically significant (p>0.05).

The blood profile of the birds fed different diets is shown in Table 3. Though, the RBCs were not significantly different, the WBCs and lymphocytes were significantly increased in chicks fed 1,3-1,6 β-glucan (p<0.05) in comparison to both chicks receiving Taylor rhzomorph and the control groups. The IgG serum peak was observed in chicks fed 2.0 g/kg 1,3-1,6 β-glucan; in particular this group showed a significantly higher IgG peak in comparison to group G1 (0.5 g/kg β-glucan) but not in comparison to group G2 (1.0 g/kg β-glucan); moreover, the IgG value observed for group G1 was significantly higher (p<0.05) in comparison to the groups T and C. Finally, no differences were observed in relation to IgM and IgA serum titer (p>0.05).

Effects of the dietary 1,3-1,6 β-glucan supplementations on relative visceral-organ weight are shown in Table 3. The relative weight of liver and spleen was not affected by dietary 1,3-1,6 β-glucan supplementations (p>0.05). For a contrary, the relative weight of bursa of Fabricius was significantly higher in the chicks fed 0.5 g/kg (G1) and 1.0 g/kg (G2) β-glucan in comparison to the positive (T) and negative (C) control group; moreover, the relative weight of bursa of Fabricius was higher (p<0.05) in group G3 (2.0 g/kg β-glucan) than in the negative control group (C).

In relation to the thickness of the intestinal wall and muscle tissue (Table 4), no significant differences were observed among the considered groups (p>0.05). For a contrary, the villus height of the chicks fed 0.5, 1.0, and 2.0 g/kg 1,3-1,6 β-glucan (group G1, G2, and G3, respectively) was higher (p<0.05) than that of the other considered groups (T and C); only exception was the villus height of group G1 at jejunum level, that was different (p<0.05) only in comparison to the positive control group (T) and not in comparison to the negative control group (C).

Similarly, the highest crypt depth and number of goblet cells were again observed for the chicks fed 1,3-1,6 β-glucan, in comparison to the other considered groups; in this case, only exception was observed for group G1 at duodenum level, with a crypt depth significantly lower than group G2 and G3, but not different in comparison to the control groups (T and C).

In relation to the number of goblet cells, at duodenum level the groups fed 1 and 2.0 g/kg β-glucan (G2 and G3, respectively) showed significantly higher values (p<0.05) in comparison to the groups G1, T, and C. Similar differences (p<0.05) were observed at jejunum level even though the difference between group G1 and G2 (0.5 and 1.0 g/kg β-glucan) was not statistically significant in comparison to the group fed the lowest β-glucan dose (0.5%; p>0.05). Finally, at ileum level, the highest goblet cell density was observed for the chicks fed 2.0 g/kg β-glucan; this value was significantly different in comparison to the group fed 0.5 g/kg (G1) β-glucan but not in comparison to the group fed 1.0 g/kg (G2) β-glucan; moreover, this latter group showed a higher (p<0.5) goblet cells density than the groups fed no β-glucan (T and C).

## DISCUSSION

Results of the present study clearly suggest that growth performances are improved when 1,3-1,6 β-glucan are included in the diet of Haidong chicks, in, particular, a beneficial effect was observed for BW, BWG, and FCR; similar results have been reported by several authors, also in relation to survival rate [8,13,17–20]. The exact mechanisms for these effects are still not completely clear; surely, the energetic contribution of the β-glucans is negligible while it is assumed that they contribute by improving innate immunity in birds [21].

Although, Zhang et al [22] study showed that there were no differences in RBC, WBC, lymphocyte in broilers fed β-glucan, Petravić-Tomenac et al [23] and Jacob and Pescatore [6] reported that an activation of WBCs such as macrophages, granulocytes and monocytes was observed in chickens fed β-glucan; In the present study, WBCs and lymphocyte also increased with 1,3-1,6 β-glucan in diet. These cells are responsible for defense against infection and support the repair of damaged tissues in the body. Dalmo and Bøgwald [24] studied the interaction between WBCs, which are in the front line of the innate immune defenses, and β-glucan; in practice, this mechanism is based on the binding ability of pattern recognition receptors (PRRs) expressed on the WBC membrane (notably on macrophages), and pathogen associated molecular patterns (PAMPs), molecular pattern that are exposed on invading pathogens cell membrane. Hence, 1,3-1,6 β-glucan act as PAMPs and once they are bound to PRRs, immune-cells get activated producing a cascade of immune-mediators, with that enhancing the immune system.

Zhang et al [18] found that the significantly higher concentration of IgG was observed on both day 21nd and day 42nd of chicken fed β-glucan. In the current study, the diet inclusion of 1,3-1,6 β-glucan significantly increased the IgG serum titer. These results suggest that 1,3-1,6 β-glucan might be used to boost the antibody production after the injection of vaccines or even as an adjuvant in the vaccine formulation; nonetheless, this hypothesis have been already demonstrated by several studies already carried out [10,11,17].

In relation to relative organs weight, many experts refer that β-glucans increase weight of immune-organs; Zhang et al [18] demonstrated that the relative weight of thymus, bursa and spleen increased when broiler chickens were supplemented with 50 to 75 mg/kg 1,3-1,6 β-glucan. Petravić-Tomenac et al [23] and Jacob and Pescatore [6] reported that β-glucan stimulates the production of precursor cells in bone marrow, resulting in an increased flow of new immunocytes into the various lymphoid organs throughout the body. In the present experiment, similar results were observed only for the bursa of Fabricius whose relative weight increased in birds fed 1.0 g/kg β-glucan, while liver and spleen relative weight were not affected. These different results may be due to the age of animals, environmental stress factors, species composition, viability, and others.

Assessment of intestinal morphology and histology is considered an important aspect that may affect dietary nutrient adsorption and also represent a primary defense barrier to pathogen [25]; also, it may be strongly affected by diet characteristics such as feed particle size and composition [26,27]. Despite in the present study no differences were observed for the thickness of intestinal wall and the thickness of muscle of wall, significant differences were observed in relation to villus heights, crypt depth and goblet cell density when β-glucan were administrated to chicks and several authors have described similar results. Solis de los Santos et al [28] found that ileum villus height, surface area, lamina propria thickness, crypt depth, and goblet cell density were enhanced as a consequence of Alphamune (yeast extract) dietary administration. Also, Morales-Lópezh et al [14] refer that chickens fed YCW and purified 1,3-1,6 β-glucan showed an increased villi height of jejunal mucosa with respect to chickens fed a control diet. Fasina and Olowo [26] reported that morphometric differences were observed in the jejunum and ileum when diet was supplement with β-glucan, while no differences were observed in duodenum. Shao et al [3] observed that dietary yeast β-glucan administration to chicks resulted in higher villus height and villus height to crypt depth ratio in comparison to chicks fed no β-glucan in jejunum. The crept in the intestinal epithelium is proliferating compartment that gives rise to cell undergoing differentiation and maturation on the villus. A deeper crept is indicative of a faster tissue turnover and perhaps, a higher demand for new tissue [26].

Finally, goblet cells in the intestinal mucosa secrete mucus which provides the first line of defense against intestinal injury [15,29]. According to the observations of Shao et al [3], the dietary β-glucan administration significantly increases the goblet cells density at jejunum level. Similarly, in the present study, villous height, crypt depth and goblet cells density of small intestine increased as a consequence of the feed supplemention with 1,3-1,6 β-glucan.

## CONCLUSION

In conclusion, the present study showed that 1,3-1,6 β-glucan dietary supplementation have significant and positive effects on growth performance, immunity indices and intestinal morphology and histology of Haidong chicks. Therefore, this practice may represent a valid tool for farmers and feed industry in the attempt of reducing the use of antibiotics in the poultry food chain. Hence, for enhancing chicks growth performances and welfare, it is suggested to include 1,3-1,6 β-glucan in poultry diet at a rate ranging between 1.0 g/kg and 2.0 g/kg.

## Figures and Tables

**Table 1:** Diets composition

Items	0.5 g/kg β-glucan	1.0 g/kg β-glucan	2.0 g/kg β-glucan	0.2 g/kg Taylor rhzomorph	Control
Ingredients (%)					
Maize	60.7	60.7	60.7	60.7	60.7
Soybean meal	25	25	25	25	25
Cottonseed meal	3	3	3	3	3
Rapeseed meal	2.96	2.96	2.96	2.96	2.96
Wheat bran	3.95	3.90	3.80	3.98	4
Limestone	1.12	1.12	1.12	1.12	1.12
Calcium hydrogen phosphate dihydrate	1.92	1.92	1.92	1.92	1.92
Sodium chloride	0.3	0.3	0.3	0.3	0.3
Vitamin premix1)	0.5	0.5	05	0.5	0.5
Mineral premix1)	0.5	0.5	05	0.5	0.5
1,3-1,6 β-glucan	0.05	0.1	0.2	0	0
Taylor rhzomorph	0	0	0	0.02	0
Calculated analysis					
ME (kcal/kg)	2,782.97	2,782.32	2,781.02	2,783.36	2,783.62
Dry matter (%)	89.12	89.08	88.96	89.15	89.17
CP (%)	21.04	21.02	20.89	21.12	21.12
Lysine (%)	0.943	0.943	0.943	0.944	0.944
Methionine (%)	0.312	0.312	0.312	0.312	0.312
Ca (%)	0.916	0.918	0.918	0.919	0.919
Average P (%)	0.506	0.506	0.506	0.506	0.506

ME, metabolizable energy; CP, crude protein.

1) Vitamin premix and Mineral provided per kilogram of diet: vitamin A, 15,000 IU; vitamin D_3_, 3,000 IU; vitamin E, 26 IU; vitamin K, 2.5 mg; vitamin B_1_, 2.1 mg; vitamin B_2_, 5 mg; vitamin B_6_, 3 mg; vitamin B_12_, 6 mg; folic acid, 1 mg; biotin, 50 μg; niacin, 24 mg; cholinechloraid, 50 mg, manganeze, 45 mg, iron, 83mg; zinc, 10 mg, copper, 0.26 mg; iodine, 1.5 mg; selenium, 1.2 mg.

**Table 2:** Effect of 1,3-1,6 β-glucan on growth performances

Items	0.5 g/kg β-glucan	1.0 g/kg β-glucan	2.0 g/kg β-glucan	0.2 g/kg Taylor rhzomorph	Control	SEM	p-value
0 to 21 d							
BW (g)	118.1^a^	122.3^a^	126.5^a^	124.4^a^	109.9^b^	4.221	0.036
BWG (g)	77.6^a^	81.8^a^	87.7^a^	84.9^a^	60.7^b^	2.962	0.028
FI (g)	144.6^a^	146.5^a^	160.4^a^	153.7^a^	114.7^b^	5.418	0.031
FCR	1.86	1.79	1.83	1.81	1.89	0.073	0.267
Mortality rate (%)	3.96	3.33	2.64	2.76	4.62	0.141	0.906
22 to 42 d							
BW (g)	282.2^a^	287.5^a^	296.6^a^	288.1^a^	241.5^b^	11.385	0.009
BWG (g)	164.1^a^	165.2^a^	170.1^a^	163.7^a^	141.6^b^	6.3116	0.018
FI (g)	439.9^a^	428.2^a^	445.1^a^	435.6^a^	392.9^b^	14.243	0.012
FCR	2.68^ab^	2.59^a^	2.61^a^	2.66^ab^	2.77^b^	0.056	0.037
Mortality rate (%)	2.42	3.05	2.34	2.35	3.64	0.137	0.329

SEM, standard error of the mean; BW, body weight; BWG, body weight gain; FI, feed intake; FCR, feed–conversion ratio.

a,b Means within rows with different superscript letters are different per p<0.05.

**Table 3:** Effect of 1,3-1,6 β-glucan on blood profiles and the visceral-organ size (% of body weight) of Haidong chicks

Items	0.5 g/kg β-glucan	1.0 g/kg β-glucan	2.0 g/kg β-glucan	0.2 g/kg Taylor rhzomorph	Control	SEM	p-value
WBC (10^5^/mm^3^)	341.4^a^	336.3^a^	338.2^a^	308.4^b^	316.1^b^	9.202	0.035
RBC (10^6^/mm^3^)	2.66	2.64	2.65	2.49	2.54	0.071	0.172
Lymphocyte (%)	77.1^a^	78.0^a^	81.0^a^	56.3^b^	58.8^b^	0.065	0.001
IgG (mg/dL)	1.66^b^	1.83^ab^	1.98^a^	1.02^c^	1.52^c^	0.041	0.008
IgM (ug/mL)	264.1	266.3	276.1	262.3	263.2	7.482	0.235
IgA (ug/mL)	279.5	285.3	278.1	275.7	268.6	7.565	0.419
Liver	2.29	2.38	2.41	2.32	2.28	0.061	0.185
Spleen	0.14	0.16	0.15	0.13	0.14	0.003	0.238
Bursa of Fabricius	0.25^a^	0.24^a^	0.23^ab^	0.19^bc^	0.18^c^	0.002	0.019

SEM, standard error of the mean; WBC, white blood cell; RBC, red blood cell; Ig, immunoglobulin.

a–c Means within rows with different superscript letters are different per p<0.05.

**Table 4:** Effect of 1,3-1,6 β-glucan on the intestinal wall thickness, intestinal muscle thickness, the height of intestinal villus, depth of crypt, and goblet cells density of Haidong chicks

Items	0.5 g/kg β-glucan	1.0 g/kg β-glucan	2.0 g/kg β-glucan	0.2 g/kg Taylor rhzomorph	Control	SEM	p-value
Intestinal wall thickness (μm)							
Duodenum	336.7	349.3	348.6	338.6	346.5	9.778	0.169
Jejunum	346.8	334.8	336.6	342.6	340.7	10.341	0.283
Ileum	338.8	339.6	329.4	332.6	331.5	10.223	0.085
Intestinal muscle thickness (μm)							
Duodenum	242.3	241.3	246.7	244.3	249.2	6.451	0.354
Jejunum	136.7	131.3	130.8	137.6	138.9	4.019	0.402
Ileum	142.5	137.6	130.5	136.8	132.4	3.971	0.817
Height of villus (μm)							
Duodenum	1,363^a^	1,386^a^	1,395^a^	1,301^b^	1,298^b^	32.342	0.027
Jejunum	1,121^b^	1,201^a^	1,212^a^	986^c^	1,089^bc^	34.316	0.001
Ileum	604.5^a^	623.4^a^	612.9^a^	521.3^b^	530.8^b^	17.335	0.004
Depth of crypt (μm)							
Duodenum	124.3^b^	168.9^a^	173.2^a^	124.2^b^	120.9^b^	4.094	0.016
Jejunum	146.4^a^	147.3^a^	142.3^a^	112.6^b^	103.5^b^	4.037	0.319
Ileum	113.5^a^	108.7^a^	117.9^a^	89.3^b^	78.5^b^	2.905	0.005
Goblet cells density (n/mm^2^)							
Duodenum	33.4^b^	52.8^a^	55.7^a^	30.4^b^	32.1^b^	1.316	0.021
Jejunum	50.3^ab^	63.8^a^	62.2^a^	39.4^b^	40.2^b^	1.348	0.008
Ileum	75.2^bc^	98.7^ab^	104.6^a^	65.1^c^	68.3^c^	2.449	0.003

SEM, standard error of the mean.

a–c Means within rows with different superscript letters are different per p<0.05.
